# P-546. Acute COVID-19 in Hospitalized Children: The Role of Viral Co-Infections

**DOI:** 10.1093/ofid/ofaf695.761

**Published:** 2026-01-11

**Authors:** Sophia Menozzi, Jesus Vazquez, Matthew Pizzuto, Sahal Thahir, Stephanie P Schwartz, Melissa Smith

**Affiliations:** UNC School of Medicine, Chapel Hill, North Carolina; University of North Carolina at Chapel Hill, Chapel Hill, North Carolina; UNC at Chapel Hill, Chapel Hill, North Carolina; University of North Carolina at Chapel Hill, Chapel Hill, North Carolina; UNC, Chapel Hill, North Carolina; University of North Carolina, Chapel Hill, North Carolina

## Abstract

**Background:**

The presence of multiple respiratory viruses in hospitalized children has been shown to be associated with worse outcomes prior to the COVID-19 pandemic. Isolated COVID-19 infections have led to hospitalization in a subset of pediatric patients. It is unclear if viral co-infection with COVID-19 is associated with worse health outcomes compared to those with isolated COVID-19 infection in hospitalized children.

**Methods:**

This was a single center review of hospitalized children with acute COVID-19 infection from April 2020 through February 2024. Children with moderate to severe disease were compared to those with mild disease to determine if viral co-infection and other health and socio-demographic characteristics led to higher disease severity. A secondary analysis based on hospital length of stay was performed.

**Results:**

Out of 102 patients, 31 had moderate to severe disease and 22 had viral co-infection (COVID-19 plus and an additional viral infection). Overall, the median hospital length of stay was 4 days and the number alive at discharge was 98 (96%). The most common co-infection virus was rhinovirus, seen in 12 of the 22 instances of co-infection. Children with COVID-19 and one additional viral infection had 1.47 (95% Confidence Interval: 0.52-3.99; p=0.46) times the odds of moderate to severe illness compared to those with only COVID-19. Non-Hispanic children had 2.74 (1.04, 8.18; p=0.04) times the odds of moderate to severe illness when compared to Hispanic children, however, on the adjusted analysis this association was no longer significant. Longer hospital stays were observed in children with moderate to severe disease, comorbidities, and males. Additionally, a non-linear relationship between hospital length of stay and age was observed.

**Conclusion:**

Disease severity and length of stay in hospitalized children with acute COVID-19 infections were not found to be exacerbated by the presence of co-infection. However, both outcomes varied by health and sociodemographic characteristics.

**Disclosures:**

All Authors: No reported disclosures

